# Large language models to develop evidence-based strategies for primary and secondary cardiovascular prevention

**DOI:** 10.1093/ehjdh/ztaf085

**Published:** 2025-08-14

**Authors:** Giulia Lorenzoni, Camilla Zanotto, Anna Sordo, Alberto Cipriani, Martina Perazzolo Marra, Francesco Tona, Daniele Gasparini, Dario Gregori

**Affiliations:** Unit of Biostatistics, Epidemiology and Public Health, Department of Cardiac, Thoracic, Vascular Sciences and Public Health, University of Padova, Padova, Italy; Unit of Biostatistics, Epidemiology and Public Health, Department of Cardiac, Thoracic, Vascular Sciences and Public Health, University of Padova, Padova, Italy; Unit of Biostatistics, Epidemiology and Public Health, Department of Cardiac, Thoracic, Vascular Sciences and Public Health, University of Padova, Padova, Italy; Cardiology Unit, Department of Cardiac, Thoracic, Vascular Sciences and Public Health, University of Padova, Padova, Italy; Cardiology Unit, Department of Cardiac, Thoracic, Vascular Sciences and Public Health, University of Padova, Padova, Italy; Cardiology Unit, Department of Cardiac, Thoracic, Vascular Sciences and Public Health, University of Padova, Padova, Italy; Unit of Biostatistics, Epidemiology and Public Health, Department of Cardiac, Thoracic, Vascular Sciences and Public Health, University of Padova, Padova, Italy; Unit of Biostatistics, Epidemiology and Public Health, Department of Cardiac, Thoracic, Vascular Sciences and Public Health, University of Padova, Padova, Italy

**Keywords:** Acute myocardial infarction, Primary prevention, Secondary prevention, Large language models, Education

## Abstract

**Aims:**

Cardiovascular diseases are the leading global cause of mortality, with ischaemic heart disease contributing significantly to the burden. Primary and secondary prevention strategies are essential to reducing the incidence and recurrence of acute myocardial infarction. Healthcare professionals are no longer the sole source of health education; the Internet, including tools powered by artificial intelligence, is also widely utilized. This study evaluates the accuracy and the readability of large language model (LLM)-generated information on cardiovascular primary and secondary prevention.

**Methods and results:**

An observational study assessed LLM’s responses to two tailored questions about acute myocardial infarction risk prevention. The LLM used was ChatGPT (4o version). Expert cardiologists evaluated the accuracy of each response using a Likert scale, while readability was assessed with the Flesch Reading Ease Score (FRES). ChatGPT-4o provided comprehensive and accurate responses for 15 out of 20 (75%) of the items. Readability scores were low, with median FRES indicating that both primary and secondary prevention content were difficult to understand. Specialized clinical topics exhibited lower accuracy and readability compared to the other topics.

**Conclusion:**

The current study demonstrated that ChatGPT-4o provided accurate information on primary and secondary prevention, although its readability was assessed as difficult. However, clinical oversight still remains critical to bridge gaps in accuracy and readability and ensure optimal patient outcomes.

## Introduction

More than half a billion people worldwide are affected by cardiovascular diseases (CVDs), which cause about a third of all global deaths. The most recent available data show that almost 40% of premature deaths from non-communicable diseases are caused by CVDs and that ischaemic heart disease is the leading cause.^1^ Several risk factors are associated with myocardial infarction risk, some modifiable, e.g. dietary patterns, and others not modifiable, such as family history. Educating people on primary and secondary prevention strategies based on well-known risk factors can reduce the risk of CVDs and improve their prognosis.

Nowadays, healthcare professionals are not the only information source for health education; the Internet has also changed how people access information, including medical advice. In the past two years, another digital innovation has enhanced how information is transmitted: the widespread adoption of large language models (LLMs).^2^ Large language models are deep-learning artificial intelligence (AI) systems that can understand user queries or commands and create various types of content (e.g. summaries, translations, texts, and images). Large language models have been rapidly adopted in many contexts, even in healthcare settings, for various tasks (e.g. analysing medical records, suggesting treatment options, education, and research).^2^

Thanks to their user-friendly nature, chat-based LLMs hold great promise in patient education, offering tailored information to support primary and secondary prevention efforts. By integrating AI into preventive strategies, healthcare systems can shift from reactive to proactive care, enhancing efficiency and overall health system performance.^3^ There is still limited medical research on the adoption of chat-based LLMs in cardiology; however, interest in this area has increased in recent months.

Considering the growing focus on the development and application of AI, even in the healthcare sector, and given that, as previously outlined, CVDs are the leading causes of death, this study aims to investigate how LLMs can or cannot be considered a resource in the prevention of CVDs. This study examines the accuracy and the readability of chat-based LLM, i.e. ChatGPT, information regarding primary and secondary cardiovascular prevention related to acute myocardial infarction.

## Methods

This study was performed between September and November 2024. Two questions regarding cardiovascular prevention in acute myocardial infarction were developed, one focusing on primary and the other on secondary prevention. The chat-based LLM chosen for the task was ChatGPT because of its widespread popularity and accessibility. The ChatGPT-4o version was employed, available from OpenAI through a ChatGPT Plus subscription. Every question was submitted once using the ‘new chat’ option. The questions were submitted to ChatGPT-4o, asking it to respond as an expert cardiologist according to the most recent guidelines. ChatGPT-4o was asked to structure the answers around the key points of primary and secondary prevention.

Assessment of the accuracy of ChatGPT-4o’s responses was conducted independently by two expert cardiologists, with a third reviewer resolving any conflict. In line with similar studies, the accuracy of each item presented in the ChatGPT-4o’s answers was evaluated with a Likert scale. For every single item of both of the answers, accuracy was graded using the following scale: (1) comprehensive: complete and accurate response; (2) correct but inadequate: all information is correct, but not complete; (3) some correct and some incorrect: information is partially correct and partially not correct; and (4) completely incorrect: all information is not correct.

Each item was also evaluated for readability with the Flesch Reading Ease Score (FRES). Flesch Reading Ease Score refers to the degree of readability of a text through a specific formula that uses syllables per word and words per sentence to calculate the final score. Flesch Reading Ease Score is based on a ranking scale (0–100) where the highest score corresponds to easy text understanding. Readability scores from 100 to 90 indicate very easy text, 89 to 80 denote easy text, 79 to 70 signify fairly easy text, 69 to 60 represent standard (plain English), 59 to 50 suggest fairly difficult text, 49 to 30 denote difficult text, and 29 to 0 correspond to very difficult text.^4^ Readability was calculated using a web-based app (https://readabilityformulas.com/).

Statistical analyses were performed using Jamovi software (version 2.3.28.0), an open-source statistical platform. Descriptive statistics were reported as absolute numbers (percentages) for categorical variables and median (I–III quartiles) for continuous variables.

## Results

**Figure 1:**
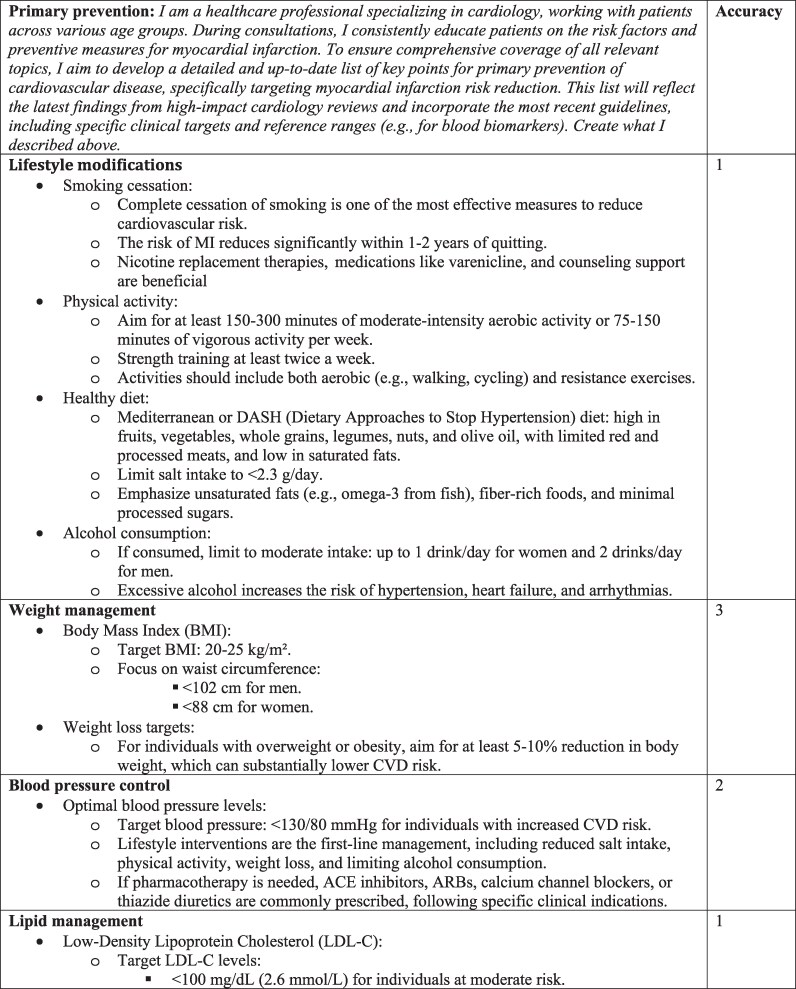

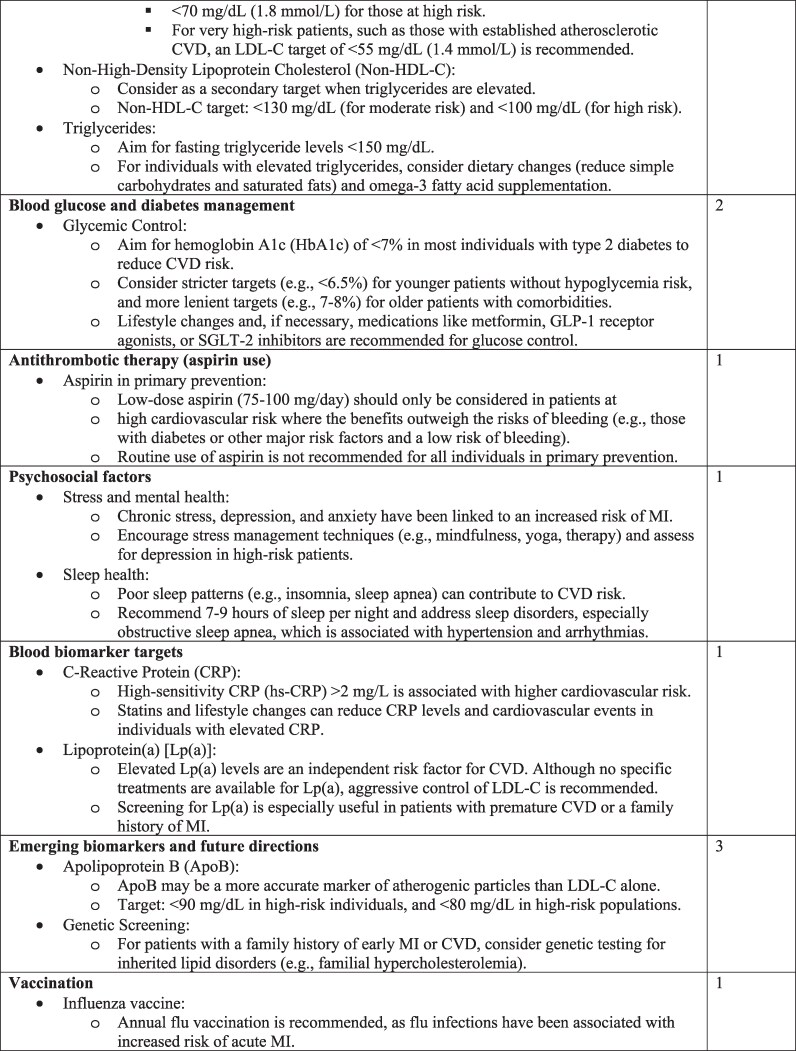

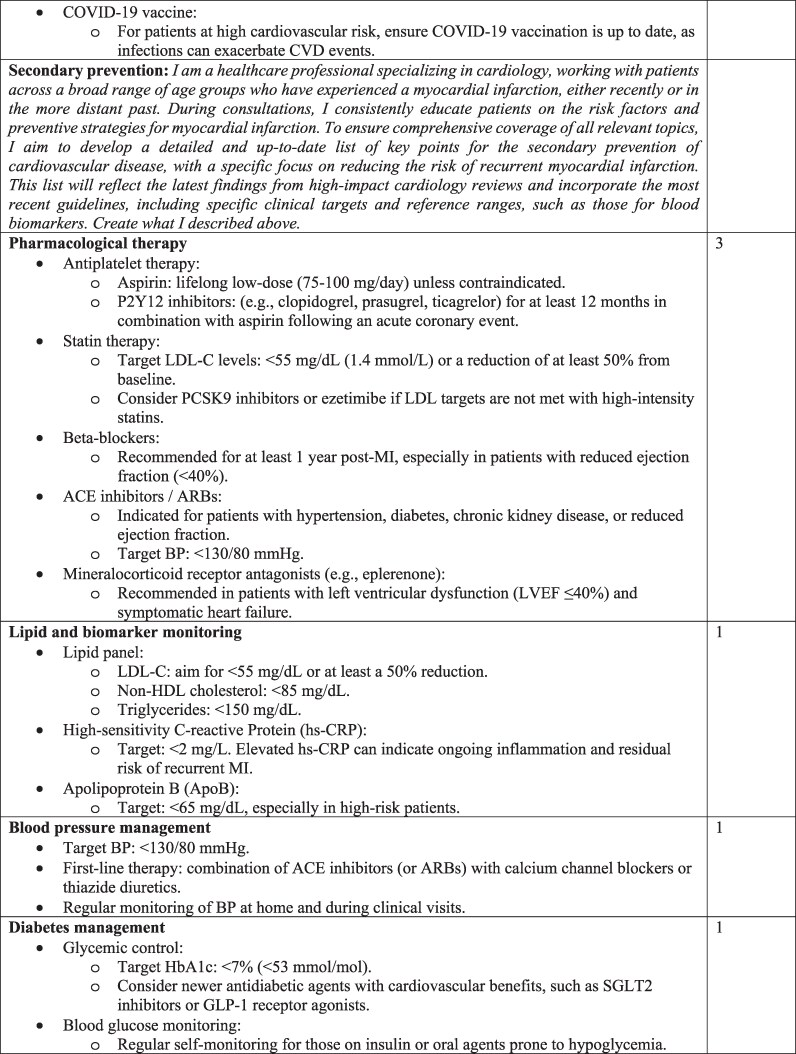

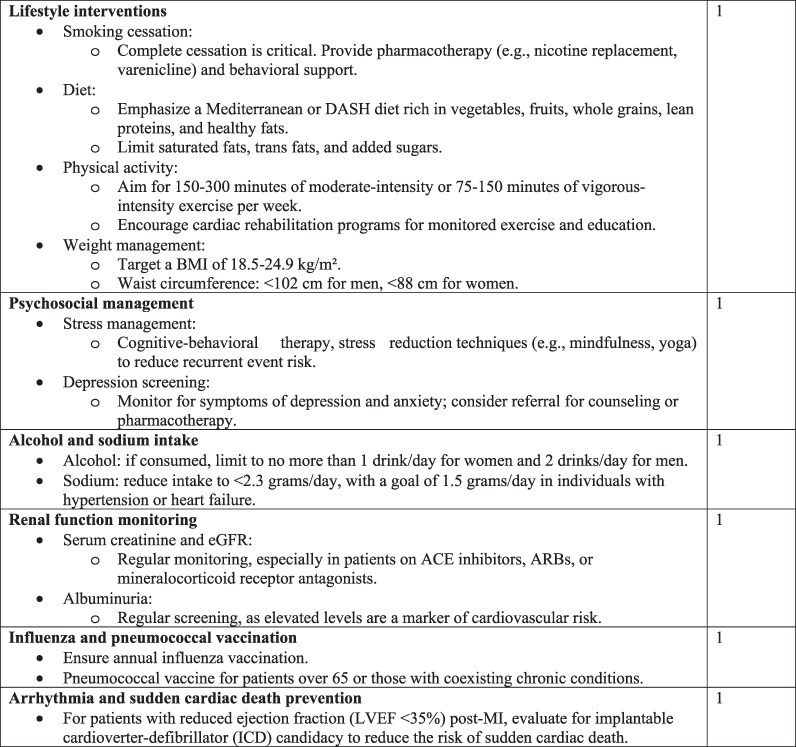
ChatGPT-4o’s answers and accuracy evaluation.

The answer for primary prevention was developed on ten key points (lifestyle modification, weight management, blood pressure control, lipid management, blood glucose and diabetes management, antithrombotic therapy, psychosocial factors, blood biomarker targets, emerging biomarkers and future directions, vaccination); the answer for secondary prevention was framed around ten key items (pharmacological therapy, lipid and biomarker monitoring, blood pressure management, diabetes management, lifestyle interventions, psychosocial management, alcohol and sodium intake, renal function monitoring, influenza and pneumococcal vaccination, arrhythmia and sudden cardiac death prevention) (*Figure 1*).

Focusing on accuracy (*Table 1*), ChatGPT-4o provided ‘comprehensive’ information (score 1) for 15/20 (75%) of the items, as judged by expert clinicians. Of the 15 items identified as ‘comprehensive,’ six were related to primary prevention, while nine addressed secondary prevention. Two items (10%) related to primary prevention were evaluated as ‘correct but inadequate’ (score 2), one regarding blood pressure control and blood glucose and diabetes management. On the other side, three items (15%), two related to primary prevention (weight management and emerging biomarkers and future directions) and one related to secondary prevention (drug therapy), were identified as ‘some correct and some incorrect’ (score 3). None of the items were evaluated as ‘completely incorrect’ (score 4).

**Table 1:** Answers accuracy and readability

	*n*	Primary prevention (*n* = 10)	Secondary prevention (*n* = 10)
Accuracy	20		
Comprehensive		6 (60%)	9 (90%)
Correct but inadequate		2 (20%)	
Some correct, some incorrect		2 (20%)	1 (10%)
Readability	20		
FRES		40 (38.3–48.8)	30.5 (11.0–46.8)
FRES frequencies			
Fairly easy		1 (10%)	
Standard			2 (20%)
Fairly difficult		2 (20%)	
Difficult		6 (60%)	3 (30%)
Very difficult		1 (10%)	5 (50%)

Data are absolute numbers (percentages) for categorical variables and median (I–III quartiles) for continuous variables.

Regarding the readability of the responses provided by the AI chatbot, the median FRES calculated was 40/100 for the primary prevention items and 30.5/100 for the secondary prevention items. These scores highlighted that both responses were considered ‘difficult text’; notably, 90% of the primary prevention items and 80% of the secondary prevention items were considered more challenging to read than standard English. Results are shown in *Table 1*.

## Discussion

The present study showed that ChatGPT-4o provides reliable general lifestyle tips for cardiovascular prevention, but proper medical supervision is still needed when it comes to specialized or patient-specific advice.

The growing adoption of AI in healthcare extends beyond patient education to applications such as clinical decision support tools. Although our study focused on the accuracy and readability of LLM-generated information for cardiovascular prevention, these aspects are also critical for the potential integration of AI models into clinical decision support systems. Ensuring that AI-generated recommendations are accurate and understandable is essential for safe implementation in clinical practice. Future research should further explore the role of LLMs in decision support, particularly in specialized areas such as cardiovascular disease management.

However, AI skills in producing highly convincing text and image-based messages, but not verified sources, raise questions about the reliability and trustworthiness of the information generated.^5^ In addition to that, limitations in accuracy and readability are emerging, underscoring the need for further improvements through professional oversight, considering LLMs opportunities and challenges for clinical research and practice.^5^

This work aims to contribute to the emerging body of literature on AI by assessing the accuracy of responses provided by ChatGPT-4o within a specific medical domain; additionally, it seeks to evaluate the readability level of the information generated. The current study demonstrated that the model provided accurate information on primary and secondary prevention, although its readability was assessed as difficult. The accuracy level described in our paper agrees with recent articles that have shown improvements for ChatGPT-4o over its predecessor ChatGPT-3.5.^6^ Another work evaluates ChatGPT-4o in acute coronary syndromes, reporting accuracy rates of at least 80%.^7^ In line with previous studies, the most accurate topics are lifestyle and exercise; on the other hand, clinical and pharmacological topics are those with worse accuracy.^8^ This could suggest a lack of ChatGPT-4o performance for more specialized clinical topics.

For what concerns readability, literature assessed the readability of the responses of ChatGPT-3 to frequently asked questions about heart failure, comparing them with educational materials from US cardiology institutions: using the FRES, it was found that information generated by ChatGPT-4o is more difficult to read compared to the content provided by cardiology societies.^9^ As emerged from our study, most of the ChatGPT-4o’s responses were considered difficult to read, with a lower FRES for the more specialized clinical topics (e.g. hypertension, renal function, and biomarkers). Notably, the items with the lowest readability scores are associated with lower accuracy levels.

## Conclusions

ChatGPT-4o shows promise as an educational resource for patients seeking general lifestyle guidance to reduce cardiovascular risk. Its accuracy is noteworthy, yet more technical or patient-specific recommendations still require careful oversight by healthcare professionals to ensure clinical appropriateness.
